# Evaluation of near-infrared spectroscopy, transcranial Doppler, and ophthalmic ultrasound measurements in critically Ill pediatric patients with increased intracranial pressure

**DOI:** 10.3389/fped.2025.1730891

**Published:** 2026-01-06

**Authors:** Damla Pinar Yavas, Dincer Yildizdas, Merve Misirlioglu, Faruk Ekinci, Faruk Incecik, Ozden Ozgur Horoz

**Affiliations:** 1 Department of Pediatric Intensive Care, Cukurova University Faculty of Medicine, Adana, Türkiye; 2 Department of Pediatric Neurology, Cukurova University Faculty of Medicine, Adana, Türkiye

**Keywords:** central retinal artery, Doppler indices, NIRS, optic nerve sheath diameter, pediatric, pulsatility index, resistive index, transcranial Doppler

## Abstract

**Objectives:**

Non-invasive methods are needed to rapidly assess increased ICP, especially for managing patients when invasive devices are unavailable or contraindicated. This study aims to examine the diagnostic value of transcranial Doppler (TCD), ophthalmic ultrasound measurements, and near-infrared spectroscopy (NIRS) to define non-invasive ICP (nICP) in the evaluation of pediatric intensive care unit (PICU) patients with increased ICP.

**Methods:**

This is a Single center prospective case-control study. The study group comprised 32 pediatric patients with increased ICP, while the control group comprised 64 healthy children. The following non-invasive methods were measured prospectively: optic nerve sheath diameter (ONSD)-derived nICP (nICPONSD), central retinal artery Doppler indices, arterial TCD blood velocities, pulsatility index (PI)-derived nICP (nICPPI), Lindegaard ratio (L/R), and NIRS values.

**Results:**

Mean ONSD, retinal artery resistive index (RI), middle cerebral artery (MCA) RI, nICPONSD, and L/R were significantly greater in the study group than the control group (*p* < 0.000, *p* < 0.000, *p* < 0.011, *p* < 0.000, *p* < 0.000, respectively). There was no significant correlation between ONSD and NIRS values or between MCA PI and NIRS values. The ONSD measurement was the strongest parameter, with an area under the curve (AUC) of 0.92 (95% CI = 0.884–0.986) and the best cut-off value being 5.27 mm (sensitivity = 76.56%; specificity = 96.87%) for detecting increased ICP.

**Conclusion:**

The availability and utilization of both TCD and ophthalmic ultrasound methods have recently increased. This is the first pediatric report that focuses on comparing ONSD, TCD, and NIRS and evaluates the Doppler indices in patients with increased ICP.

## Introduction

Increased intracranial pressure (ICP) is an important syndrome caused by numerous neurologic diseases. Monitoring and managing increased ICP in critically ill pediatric patients is vital to prevent secondary brain injury, poor neurological outcomes, and mortality (1). Invasive ICP measurement is the gold standard for initially diagnosing and monitoring increased ICP. However, it is invasive, expensive, and has contraindications, such as hepatic encephalopathy with coagulopathy, bleeding disorders, and brain abscess. It is also not always available due to a lack of equipment (2, 3). In such settings, non-invasive methods are needed to rapidly assess increased ICP and manage patients. CT and MRI have some limitations: they take a long time, necessitate patient transportation, and are expensive. Point-of-care ultrasound is a non-invasive method that might be preferable due to its low cost and rapid assessment capabilities (4). Near-Infrared Spectroscopy (NIRS) can assess changes in regional cerebral oxygen saturation and provides non-invasive monitoring of cellular tissue oxygenation (5). Elevated ICP can reduce cerebral blood flow and regional cerebral oxygen saturation, potentially leading to lower NIRS value. However, NIRS alone can not be used to calculate an indirect ICP value such as those derived from ONSD or TCD-based PI (5). The ONSD is non-invasive and can be quickly performed at the bedside. The dura mater extends along the optic nerve, which is also surrounded by cerebrospinal fluid (CSF). Increased ICP is transmitted through the subarachnoid space. Additionally, the central retinal and ophthalmic arteries' Doppler indices, such as peak systolic blood velocities (PSV), end-diastolic blood velocities (EDV), pulsatility index (PI), and resistive index (RI) measurements, can be used in cases of increased ICP (6, 7). Using TCD ultrasonography can provide a bedside assessment of cerebral blood flow velocities and rapid changes in cerebral perfusion in critically ill children with increased ICP. This technique quantifies the PSV, EDV, and mean cerebral blood flow velocities (MFV) within the large cerebral arteries. The non-invasive determination of ICP using TCD can be calculated using the Gosling PI. The PI is a hemodynamic measure calculated as the difference between the PSV and EDV divided by the mean velocity [(PSV−EDV)/MFV] (8). Cerebral vasospasm (CV) suggests hyperdynamic flow, stenotic arterial disease, or a vasospastic reaction. CV is determined when the Lindegaard ratio [ratio of flow in the MCA to the extracranial internal carotid artery (ICA); L/R] is ≥3 (9). The L/R helps differentiate hyperemia from CV. Hyperemia would result in flow elevations in both the MCA and extracranial ICA, resulting in an L/R <3. An L/R between 3 and 6 is a sign of mild vasospasm, and >6 is an indication of severe vasospasm (10).

The aim of this study is to examine the diagnostic value of TCD, ophthalmic ultrasound measurements, and NIRS in defining non-invasive ICP (nICP). We also sought to determine the possible correlation between these non-invasive methods in the evaluation of pediatric intensive care unit (PICU) patients with increased ICP. We aimed to calculate nICP using the correlated PI formula with TCD and the ONSD-derived formula with ophthalmic ultrasound.

## Materials and methods

Patients admitted to our PICU at Çukurova University Medical Faculty between May 2020 and May 2021 due to clinical or radiological suspicion of increased ICP were included in this single-center study. Clinically, suspicion of increased ICP was determined by altered consciousness level, loss of brainstem reflex, dilated or unresponsive pupil, cranial nerve injury, and Cushing's triad. Radiologically, it was identified by shifts, ventricular collapse, effacement of sulci, and compression in the cisterna (11). Data was collected prospectively from 32 children (1 month-18 years) with increased ICP. 64 children, who were admitted to our general pediatric outpatient clinic and did not have increased ICP symptoms or any other neurological diseases, were included as the control group. Exclusion criteria include being under one month or over 18 years old, having a history of ocular pathology or optic nerve trauma, absence of informed consent, skull base fracture with a CSF leak, an inaccessible ultrasound temporal window, and a diagnosis of brain death, patients who were transferred from other hospitals Demographic data, Glasgow Coma Scale (GCS) at admission, the pediatric version of Glasgow Outcome Score (GOSE PEDS) (3) at discharge, diagnosis at admission, results of cranial CT and MRI examinations, and results of the TCD, ONSD, and NIRS measurements were recorded. All initial TCD, ONSD measurements were performed within 24 h after admission to our PICU, measurements were repeated four times a day and continued until the patient was discharged or for the first seven days.

The study was approved by the Ethics Committee for Clinical Research in Çukurova University Medical Faculty. The study protocol was approved by the local institutional ethics committee and was performed in accordance with the Helsinki Declaration of 1975. Written parental consents were obtained from all participants.

### Transcranial Doppler ultrasound method

**Figure 1:**
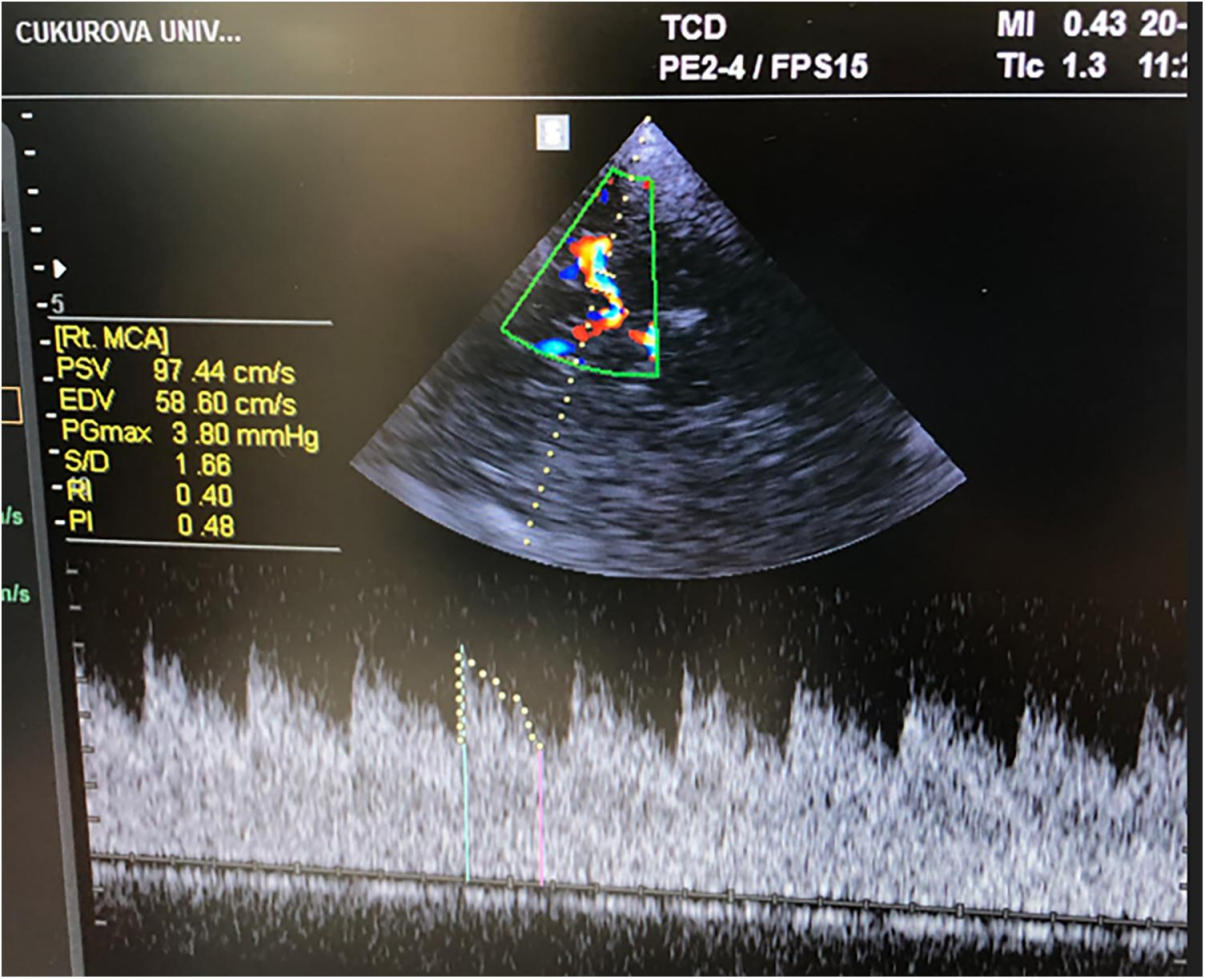
TCD MCA blood velocities and Doppler indices.

The TCD examinations were performed by two experienced PICU fellows (DPY, MM), who completed a point-of-care ultrasound (POCUS) course provided by the Turkish Pediatric Emergency and Intensive Care Society using a Mindray® Ultrasound device with a 2 MHz transducer. In training sessions prior to the study, a coefficient of <10% variation for each study measurement between sonographers was demonstrated. TCD recordings were included in the study if the bedhead was adjusted to 20°–30°; the head of the patient was positioned at the midline; arterial carbon dioxide pressure (PaCO₂) was between 30 and 45 mmHg; and hemodynamics were stable with blood pressure within the normal range for age while map values were maintained within the normal age-adjusted reference ranges, in accordance with institutional pediatric TBI protocols. For each patient, we recorded bilateral MCA flow velocities (PSV, MFV, and EDV), PI, and RI measurements using the transtemporal window (Figure 1). We used the PI and correlation formula (ICP = 10.927 × PI–1.284) to calculate nICP (nICP_PI_) (12). For L/R extracranial ICA velocity flows were recorded.

### Ophthalmic ultrasound method

**Figure 2:**
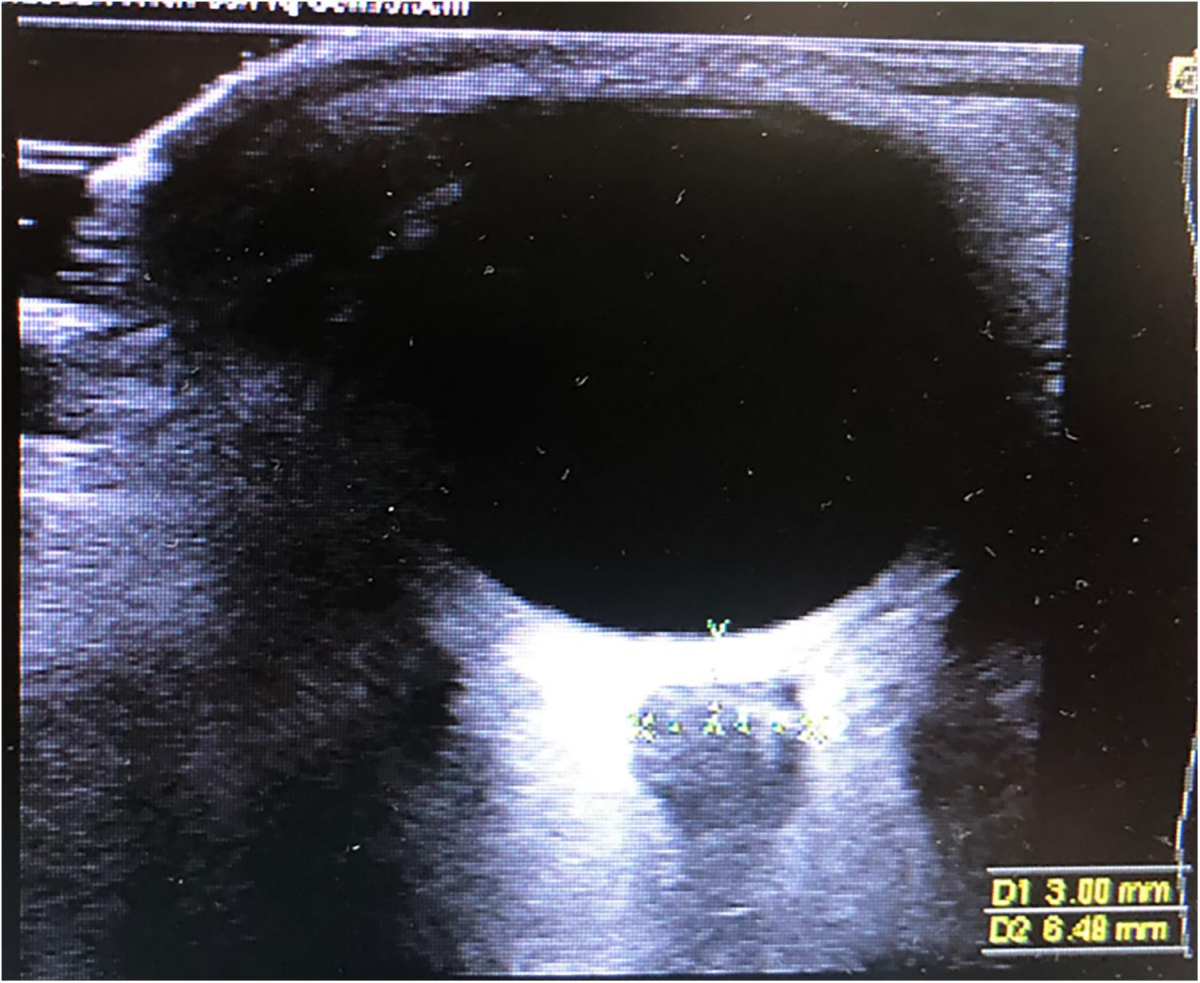
Ultrasonographic OSND measurement.

**Figure 3:**
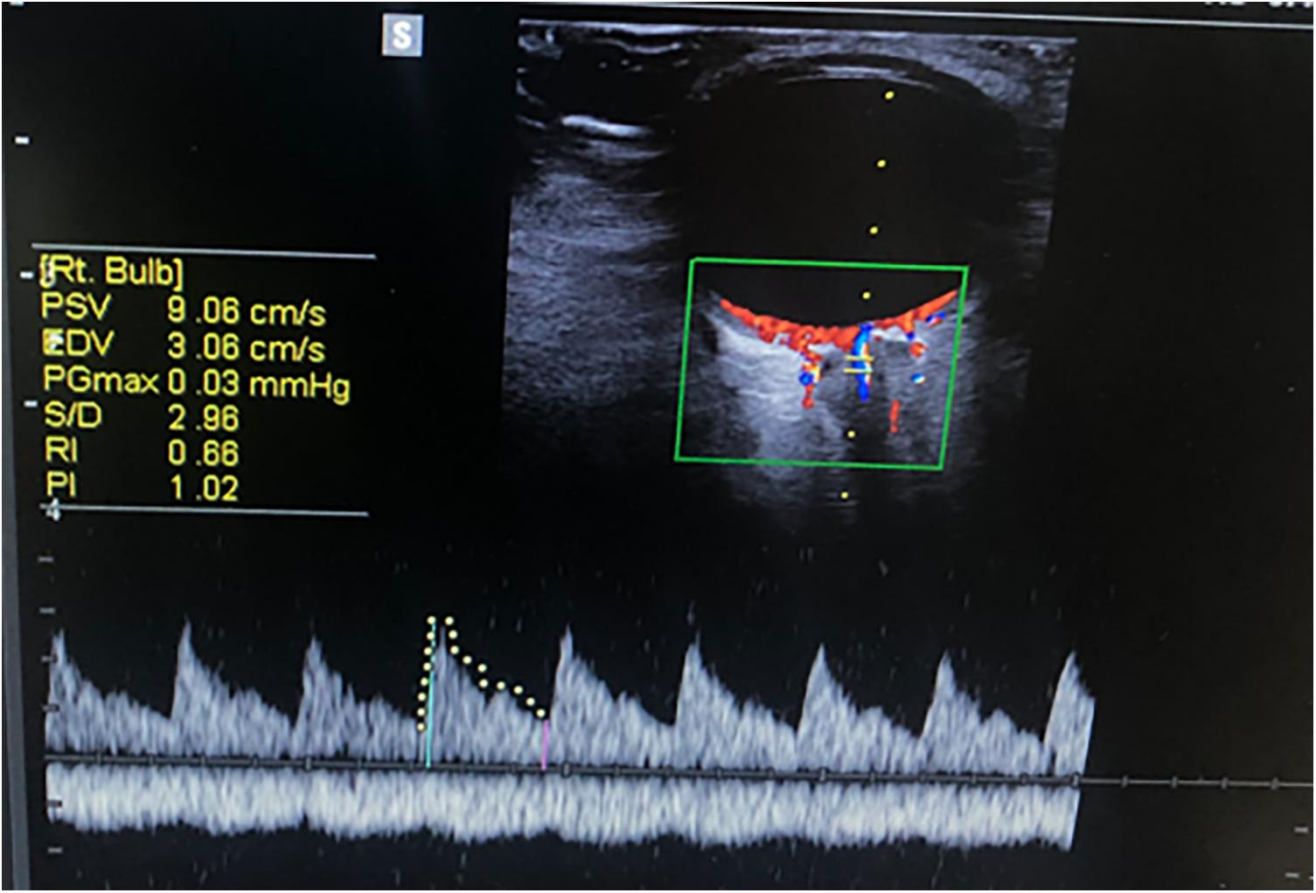
Measurement of ophthalmic artery Doppler indices.

A 7 MHz linear probe and a Mindray® Ultrasound device was used to perform ultrasound examinations of the ONSD. Measurements were made in both the axial and sagittal planes, 3 mm behind the retina in both eyes. The mean value of these measurements was calculated (Figure 2). The central retinal artery flow velocities and Doppler indices (PSV, EDV, PI, RI) were recorded with colored Doppler (Figure 3). Non-invasive ICP was calculated with ONSD (nICP_ONSD_: 5 × ONSD−13.92 mmHg) (3).

### NIRS

The INVOS® 5100C Cerebral Oximeter (Somanetics, Troy, MI) was used for the NIRS. Measurements were made using a disposable sensor placed on the bilateral frontal area of the head and continued until the patient was discharged or for the first seven days.

### Statistical analysis

The software IBM SPSS Statistics Standard 26 was used for the statistical analysis of the data. Descriptive statistics (mean, standard deviation, median, minimum, maximum, IQR) were used. The normal distribution of the numerical variables' data was evaluated with the Shapiro–Wilk test of normality. The Mann–Whitney *U*-test was used for variables that did not show a normal distribution in the comparison of study groups. Relationships between numerical variables were evaluated using the Pearson and Spearman correlation coefficients. The performance of clinical variables in predicting increased ICP was evaluated using receiver operating characteristics (ROC) curve analysis. A statistical significance level of 0.05 was implemented for all tests.

## Results

**Figure 4:**
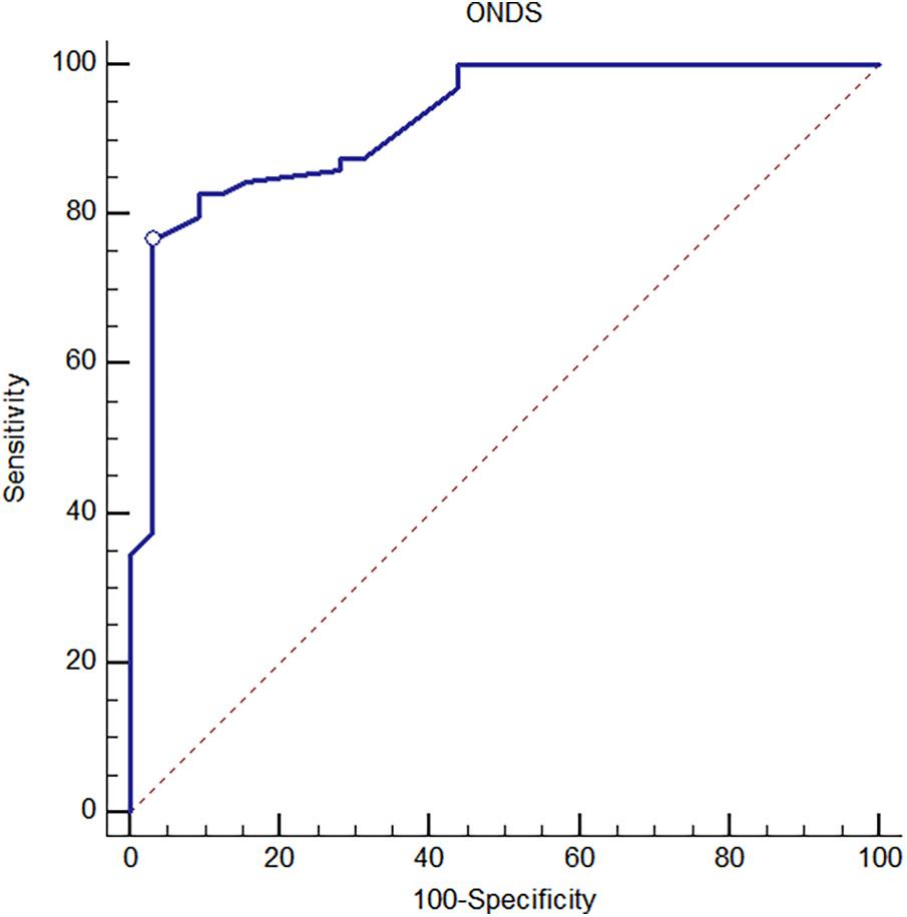
The ROC curve of ONSD for predicting increased ICP.

A total of 287 TCD and ophthalmic ultrasound measurements from 32 patients (17 females) with increased ICP and 67 measurements from 64 children (35 females) who were in the control group were analyzed. Demographic characteristics of patients and control group are given in Table 1. Thirty-one patients (97%) underwent cranial CT, and the most common diagnosis (*n* = 19) was local/generalized edema. The underlying diagnoses of increased ICP and other findings are listed in Table 2. Four patients (12.5%) died. Ultrasonographic measurements and nICP measurements of the patient and control groups are given in Table 3. Mean ONSD, retinal artery RI, MCA RI, nICP_ONSD_, and L/R were significantly greater in the study group than the control group (*p* < 0.000, *p* < 0.000, *p* < 0.011, *p* < 0.000, *p* < 0.000, respectively). Although the MCA PI values were greater in the study group than in the control group, no statistically significant value was found (*p* = 0.147) (Table 3). Table 4 shows all the parameters that were evaluated for the prediction of increased ICP; among them, the ONSD exhibited the strongest relation with its area under the curve (AUC) value of 0.92 (95% confidence interval: 0.869–0.978) (Figure 4) and the best cut-off value being 5.27 mm (sensitivity = 76.56%; specificity = 96.87%) (Table 4). There was no significant correlation between ONSD and NIRS values or between MCA PI and NIRS values. However, a significant positive relationship was observed between NIRS and both mPSV and MFV (*p* = 0.029 and 0.011, respectively).

**Table 1:** Demographic characteristics of patients and control group.

Characteristics	Patient group *n* *=* 32	Control group *n* *=* 64	*p*-value
Age (months)			
Mean ± SD	88.88 ± 56.5	111.98 ± 49.03	0.065
Median (min–max)	91.5 (5–202)	106 (4–204)	
Gender, *n* (%)			
Male	15 (47)	29 (45)	0.885
Female	17 (53)	35 (55)	
Age, *n* (%) (years)			
<1	2 (6)	1 (2)	0.115
1–5	12 (38)	13 (20)	
6–10	7 (22)	25 (39)	
>10	11 (34)	25 (39)	

**Table 2:** The underlying diagnosis of increased ICP and other findings in the patient group.

Parameter	Patient group *n* *=* 32
GCS	
Mean ± SD	9.53 ± 3.78
Median (min–max)	11 (3–14)
GCS Scoring, *n* (%)	
Mild (GCS: 12–15)	12 (38)
Moderate (GCS: 9–11)	10 (31)
Severe (GCS: 3–8)	10 (31)
PICU hospitalization day	
Mean ± SD	11.22 ± 13.82
Median (min–max)	5 (2–68)
NIRS values	
Mean ± SD	66.53 ± 14.33
Median (min–max)	62.5 (38–95)
Cranial CT findings, *n* (%)	
Subdural hematoma	2 (6)
Subarachnoid hematoma	2 (6)
Intraparenchymal hematoma	3 (9)
Epidural hematoma	1 (3)
Cerebral contusion ve laceration	2 (6)
Subgaleal hematoma	1 (3)
Cerebral infarct	2 (6)
Local/Generalized edema	19 (59)
Need for invasive mechanical ventilation, *n* (%)	
Yes	13 (41)
No	19 (59)
Diagnosis, *n* (%)	
Traumatic causes	8 (25)
Non-traumatic causes	24 (75)
Meningoencephalitis	6 (19)
Post-arrest care	4 (13)
Stroke	2 (6)
Satus	1 (3)
İntracranial mass	1 (3)
Intracranial bleeding	3 (9)
Others	7 (22)

**Table 3:** Comparison of ultrasonographic measurements and nICP measurements of the patient and control groups.

Measurement type	Patient group *n* = 32 Number of measurements = 287	Control group *n* = 64 Number of measurements = 67	*p*
	Mean ± SD	Mean ± SD	
ONSD, (mm)			
Mean ± SD	5.73 ± 0.47	4.92 ± 0.4	**0** **.** **000**
M (IQR)	5.6 (0.57)	4.9 (0.66)	
(min–max)	(4.78–7)	(4.15–5.52)	
Retinal artery RI			
Mean ± SD	0.72 ± 0.17	0.59 ± 0.08	**0** **.** **000**
M (IQR)	0.71 (0.21)	0.57 (0.1)	
(min–max)	(0.5–1.4)	(0.43–0.8)	
MCA MFV, (cm/s)			
Mean ± SD	77.16 ± 28.32	76.1 ± 13.12	0.622
M (IQR)	72.14 (47.08)	74.93 (16.75)	
(min–max)	(35.26–132.45)	(50.6–112.2)	
MCA EDV, (cm/s)			
Mean ± SD	50.08 ± 23.57	52.52 ± 12.42	0.164
M (IQR)	42.72 (39.98)	52.74 (15.87)	
(min–max)	(14.83–93.91)	(5.59–94.61)	
MCA PI			
Mean ± SD	1.05 ± 0.38	0.88 ± 0.18	0.147
M (IQR)	0.91 (0.55)	0.89 (0.25)	
(min–max)	(0.52–1.98)	(0.43–1.28)	
MCA RI			
Mean ± SD	0.63 ± 0.13	0.56 ± 0.08	**0** **.** **011**
M (IQR)	0.6 (0.17)	0.57 (0.1)	
(min–max)	(0.43–0.98)	(0.36–0.71)	
nICP_PI_			
Mean ± SD	10.2 ± 4.07	8.29 ± 1.98	0.095
M (IQR)	8.63 (5.72)	8.35 (2.65)	
(min–max)	(4.4–20.3)	(4–12.7)	
L/R			
Mean ± SD	1.89 ± 0.81	1.11 ± 0.18	**0** **.** **000**
M (IQR)	1.89 (1.43)	1.1 (0.23)	
(min–max)	(0.52–3.2)	(0.8–1.95)	
nICP_ONDS_			
Mean ± SD	14.73 ± 2.35	10.57 ± 1.93	**0** **.** **000**
M (IQR)	14 (3)	10.5 (2.96)	
(min–max)	(9.9–21)	(6.8–13.92)	
MCA PSV, (cm/s)			
Mean ± SD	120.89 ± 42.33	120.67 ± 20.37	0.568
M (IQR)	116.64 (71.08)	119.18 (25.89)	
(min–max)	(43.78–215.49)	(84.02–162.4)	

Bold values indicate highly significant *p* values (*p* < 0.05).

**Table 4:** Evaluation of ultrasound measurement results in order to predict the increased ICP by ROC curve analysis.

Measurement type	*AUC* (95% CI)	*p*-value	Youden index J	Cut-off	Sensitivity (95% CI)	Specificity (95% CI)
Retinal artery RI	0.77 (0.666–0.878)	**<0** **.** **0001**	0.48	0.64	85.94 (75.0–93.4)	62.50 (43.7–78.9)
ONSD	0.92 (0.869–0.978)	**<0** **.** **0001**	0.73	5.27	76.56 (64.3–86.2)	96.87 (83.8–99.9)
MCA RI	0.66 (0.537–0.782)	**0** **.** **0109**	0.28	0.63	90.62 (80.7–96.5)	37.50 (21.1–56.3)
MCA PI	0.59 (0.460–0.723)	0.1746	0.27	1.2	95.31 (86.9–99.0)	31.25 (16.1–50.0)
MCA EDV	0.59 (0.440–0.735)	0.2444	0.36	41.66	85.94 (75.0–93.4)	50.00 (31.9–68.1)
MCA MFV	0.53 (0.379–0.683)	0.6887	0.31	59.78	90.62 (80.7–96.5)	40.63 (23.7–59.4)
L/R	0.77 (0.644–0.906)	**<0** **.** **0001**	0.66	1.3	93.75 (84.8–98.3)	71.87 (53.3–86.3)
nICP_PI_	0.60 (0.476–0.734)	0.1101	0.30	11.9	98.44 (91.6–100.0)	31.25 (16.1–50.0)
nICP_ONDS_	0.94 (0.884–0.986)	**<0** **.** **0001**	0.77	12.4	79.69 (67.8–88.7)	96.87 (83.8–99.9)
MCA PSV	0.54 (0.389–0.683)	0.6314	0.27	98.8	85.94 (75.0–93.4)	40.63 (23.7–59.4)

Bold values indicate highly significant *p* values (*p* < 0.05).

**Figure 5:**
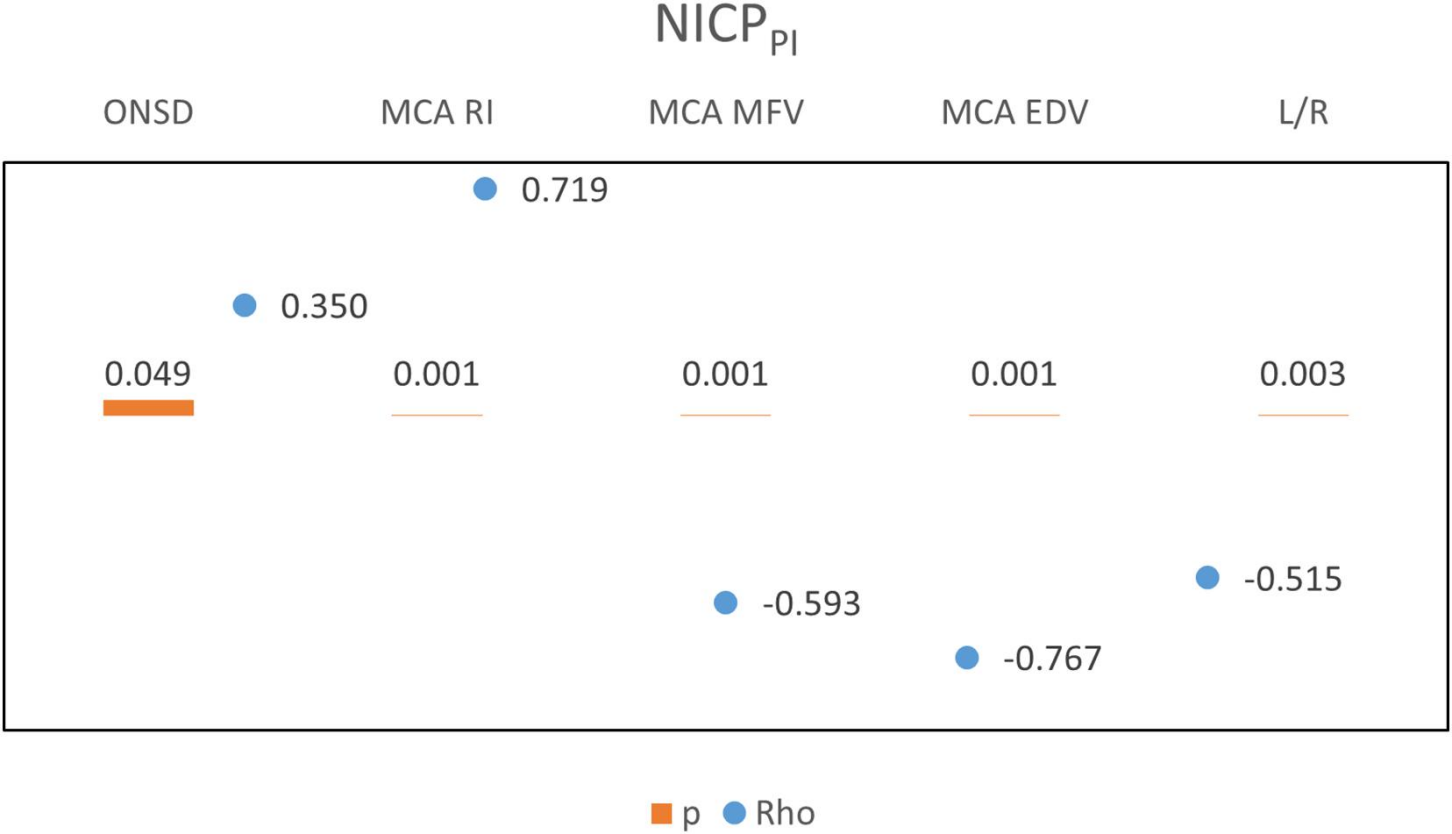
Correlations between Non-invasive ICP (nICPPI) estimates and ultrasound parameters.

**Figure 6:**
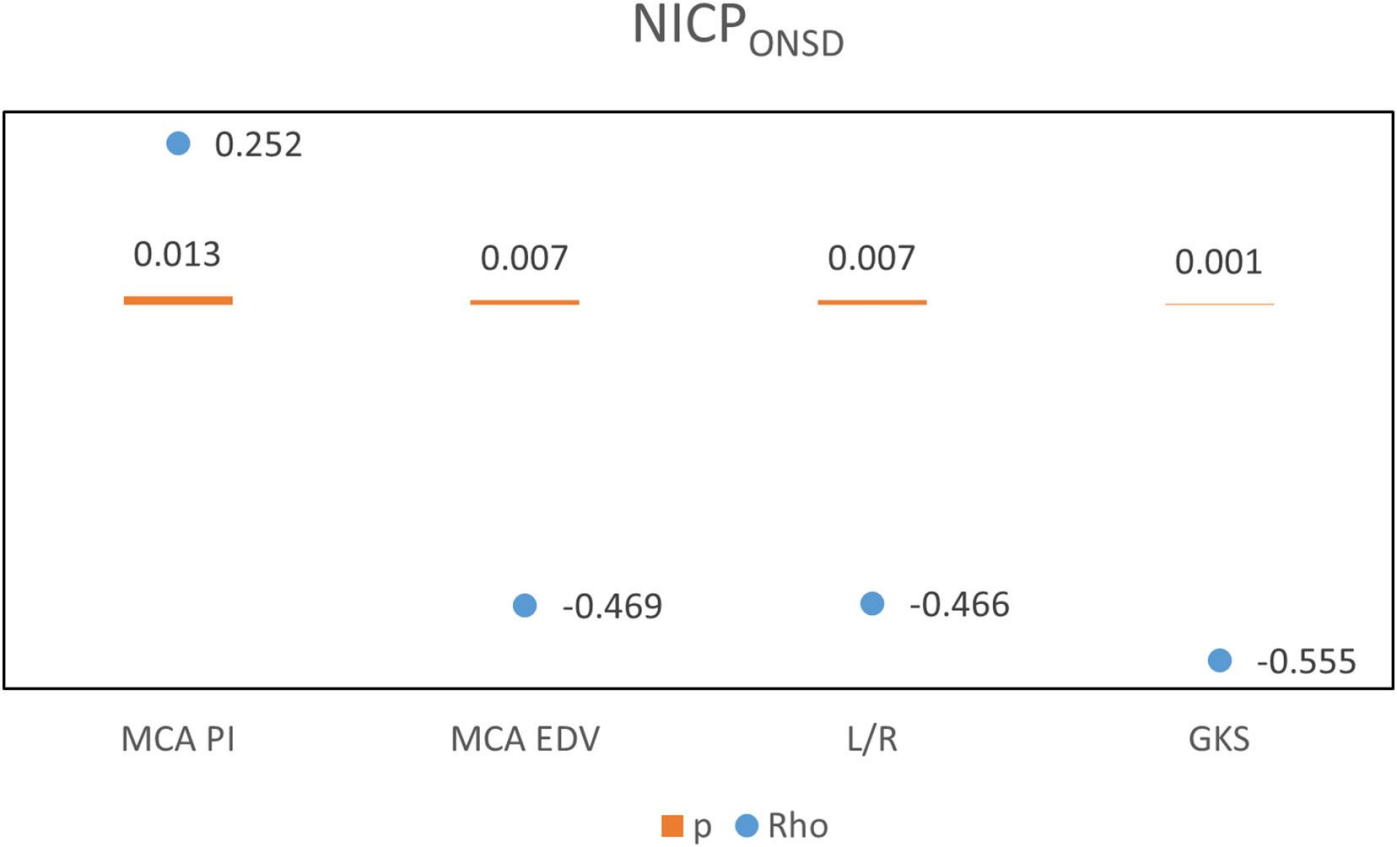
Correlations between Non-invasive ICP (nICPONSD) and ultrasonographic parameters.

A positive relationship was revealed between nICP_PI_ and ONSD, and MCA RI (Figure 5). A negative relationship was observed with MCA MFV, MCA EDV, and L/R There was a positive relation between nICP_ONSD_ and MCA PI and a negative relation with MCA EDV, L/R, and GKS (Figure 6). A significant relationship was found between L/R and the diagnoses of meningoencephalitis, post-arrest care, and non-traumatic intracranial hemorrhage.

## Discussion

According to our results, ONSD had the best correlation with increased ICP compared to TCD and NIRS. We found that, clinically suspected ICP patients had higher PI values. Several studies comparing TCD and ONSD in patients with suspected increased ICP have reported inconsistent and sometimes conflicting results (7, 13–15). Although some pediatric studies have examined NIRS together with either TCD or ONSD, no previous pediatric study has evaluated ONSD, TCD, and NIRS simultaneously within the same cohort. To our knowledge, this is the first pediatric report comparing ONSD, TCD, and NIRS together in the same cohort of children with suspected increased ICP.

The PI has been one of the most frequently used TCD-derived nICP estimation methods. Many studies in the literature focused on the association between PI and ICP in patients with increased ICP (9). Some authors have reported a strong association between PI and ICP; however, other studies have reported poor correlations between these parameters. Nakip et al. evaluated TCD-PI and ONSD together in a pediatric TBI cohort for non-invasive ICP assessment, and reported that both parameters have potential value for monitoring and prognosticanation (16). Rasulo et al. conducted a study in which 16 international centers participated and reported that nICP, calculated by TCD measurements, had high accuracy in excluding intracranial hypertension. The correlation between TCD nICP and invasively measured ICP was 33.3% (17). We similarly found that nICP_PI_ had values significantly lower than 15 mmHg in the control group, while they had different values in the study group. In this study, PI showed a weak relationship with an AUC value of 0.59 (0.440–0.735) for the prediction of increased ICP.

RI was also used to predict increased ICP. El-Maghrabi et al. performed RI measurements of the MCA with 30 infants with possible hydrocephalus. In terms of RI measurements, they found a significant difference between infants with high ICP and those with normal ICP (*p* = 0.001). The cut-off point of the RI was 0.7, with high sensitivity, specificity, and accuracy (18). In the literature, several studies have shown a good correlation between RI and raised ICP (14, 19). Likewise, we found that the RI was significantly greater in the study group than in the control group (*p* < 0.011). The cut-off value was 0.63 (sensitivity: 90.62%; specificity: 37.5%) for the prediction of increased ICP.

In the literature, studies have reported that vasospasm following head trauma contributes to poor outcomes and have highlighted the potential role of TCD in the detection of vascular spasms (15). The American Heart Association gives a class IIa recommendation to the statement, “TCD is reasonable to monitor for the development of arterial vasospasm” (20). In a study focused on 69 children with moderate and severe TBI that used L/R > 3, the incidence of vasospasm was 9% in children with moderate TBI and 34% in children with severe TBI (21). In the current study, the incidence of vasospasm (L/R > 3) was 22% (*n* = 7), while a negative relationship between L/R and increased ICP was found. We found a significant relationship between L/R and the diagnoses of meningoencephalitis, post-arrest care, and non-traumatic intracranial hemorrhage.

In a study conducted on 38 pediatric patients with increased ICP and 19 healthy children, the authors found that ONSD and RI (mean ONSD = 5.9 ± 0.8 mm, mean RI = 0.71 ± 0.08) were significantly higher in the study group than in 19 healthy children. The ONSD measurement was the strongest parameter, with an AUC value of 0.767, for predicting increased ICP (7). In our study, the central retinal artery RI (mean RI = 0.72 ± 0.17) and ONSD values were significantly higher in the study group. The best cut-off value of RI was 0.64 (sensitivity: 85.94%; specificity: 62.50%).

Padayachy et al. determined that the best ONSD cut-off values for detecting ICP ≥15 and ≥20 mmHg were 5.49 (sensitivity: 93.7%; specificity: 74.4%) and 5.75 mm (sensitivity: 88.9%; specificity: 84.2%), respectively, in 172 children who required invasive ICP monitoring (22). In our study, the mean ONSD was 5.73 ± 0.47 mm in the group with suspected increased ICP and 4.92 ± 0.4 mm in the control group. We determined the cut-off value to be 5.27 mm (sensitivity: 76.56%; specificity: 96.87%) for ONSD in the study group. In pediatric patients younger than 16 years, the study compared ONSD-derived nICP, TCD-PI–based (PIa) nICP, and TCD diastolic-velocity–based nICP methods; ONSD demonstrated the strongest correlation, and the study focused exclusively on non-invasive ICP estimation techniques (3). Our study determined that ONSD exhibited the strongest relationship with an AUC value of 0.92 (95% CI: 0.869–0.978).

In a study conducted on 47 adult patients with TBI, the authors determined a significant correlation between retinal RI and invasive ICP (23). Tarzamni et al. reported that ONSD, PSV, and RI (mean RI = 0.76 ± 0.07) were significantly higher in 30 children with raised ICP than in 30 healthy children (13).

Zuluaga et al. determined that in 30 children, who had increased ICP and underwent invasive ICP monitoring, NIRS decreased with an increase in ICP, but there was no significant correlation between NIRS and increased ICP (*p* = 0.3) (24) Pilot pediatric study that compared NIRS and ONSD measurements in children with suspected raised ICP. Found no strong correlation between NIRS and ONSD (2). In our study, no significant correlation between NIRS and increased ICP was found. On the other hand, a positive relation between NIRS values and PSV and EDV measurements was observed throughout the study.

Systematic and narrative reviews that collate pediatric and adult data for ONSD, TCD, NIRS and other non-invasive approaches—useful to cite when you argue that the pediatric evidence base is still limited and heterogeneous (25).

The major limitation of our study was the inability to routinely apply invasive ICP monitoring in our PICU, owing to the high equipment costs and the absence of an experienced neurosurgery team in our hospital. In the current study, we used clinical and radiological methods to measure an increase in ICP. Unfortunately, the correlation between invasive ICP and ONSD, TCD, and NIRS could not be determined. Another limitation is the study's small sample size. Further prospective studies with a larger number of patients are needed.

## Conclusion

Our study found that ONSD exhibited the best accuracy when compared to NIRS- and TCD-derived nICP methods for detecting increased ICP. NIRS values reflect regional cerebral oxygen saturation and show correlations with clinically suspected increased ICP, but they do not provide a quantitative ICP value.

In the literature, a limited number of studies evaluated NIRS and ONSD or TCD and ONSD in patients with increased ICP. To our knowledge, this study contributes to such limited pediatric literature evaluating ONSD, TCD, and NIRS together in patients with clinically suspected increased ICP, Further studies with larger series focusing on ultrasound methods for nICP assessment are needed.

## Data Availability

The raw data supporting the conclusions of this article will be made available by the authors, without undue reservation.
